# How to integrate youth in regional sustainability transformation processes: Tools, structures, and effects

**DOI:** 10.1007/s13280-024-02048-x

**Published:** 2024-07-02

**Authors:** Cornelia Fischer, Verena Radinger-Peer

**Affiliations:** 1 Department of Economics and Social Sciences, Institute of Sustainable Economic Development, University of Natural Resources and Life Sciences, Vienna, Feistmantelstraße 4, 1180 Vienna, Austria; 2 Department of Landscape, Spatial and Infrastructure Sciences, Institute of Landscape Development, Recreation and Conservation Planning, University of Natural Resources and Life Sciences, Vienna, Peter-Jordan-Straße 65, 1180 Vienna, Austria

**Keywords:** Integration, Participatory mapping, Participation, Real-world experiment, Real-world laboratory, Transdisciplinarity

## Abstract

This study examines young people’s involvement in regional sustainability transformation processes based on a real-world experiment in a community of 5700 inhabitants on the southern outskirts of city of Vienna, Austria. The eight-month experiment aimed to explore methods and tools for transdisciplinary co-creation with youth, the impact of structural conditions on their participation and the effects of their integration. Findings highlight the crucial roles of topics relevant to youth, a trusted intermediary like a youth worker, and structural conditions such as political support and resource allocation in enhancing youth engagement success. Collaborative decision making with policymakers and direct communication were also key to effective participation. The real-world experiment laid the groundwork for future participatory methods and had an impact on youth–community relations. It affirmed the role of youth in regional development, with effects that extended beyond the immediate scope of the experiment in terms of time, space, and topic.

## Introduction

Research on sustainability transformation governance notes a shift from consultation (e.g., in the frame of Local Agenda 21 processes, Feichtinger and Pregernig 2005) to cooperative and experimental co-creation processes (Loorbach and Rotmans 2010). Civil society actors, including diverse individuals in terms of age, gender, motivation, and educational background (Ehnert et al. 2018), play a crucial role in these transformation processes (Frantzeskaki et al. 2016). Young people,^1^ as a subset of civil society, are particularly interesting due to their openness to experimentation and new solutions, which distinguishes them from other groups of actors (Frantzeskaki et al. 2016). However, young people are considered a hard-to-reach group, especially outside institutionalized contexts like schools or associations (Bauml et al. 2022). Different models, such as Hart’s ladder of participation (1992), Shier’s five-stage model (2001), and Lundy’s model of participation (2007), offer approaches to capturing different levels of participation, however, there is no universal model of youth participation; rather, the method or level chosen must be appropriate to purpose and context (McCafferty 2017). Integrating young people’s perspectives requires a multistage process and age-appropriate methods, which are essential to maximizing their inclusion, participation, and empowerment (Mozammel and Schechter 2005; Shamrova and Cummings 2017; Kennan et al. 2019; Camponovo et al. 2023) and transparent feedback on their influence (Lansdown 2014). Recognizing young people as knowledgeable participants and full citizens is crucial for a comprehensive examination of their lived realities and a better understanding of the research subject (Botchwey et al. 2019; Moody et al. 2021; Camponovo et al. 2023). However, efforts to involve youth are often incomplete and symbolic, with additional barriers such as lack of ownership, partnerships, and supportive policies and structures (Gal 2017; Bauml et al. 2022).

Particularly concerning the latter, real-world laboratories have established promising research infrastructures that enable transdisciplinary research practice (Bergmann et al. 2021) to foster knowledge production among scientific and societal actors in real-world settings. A real-world laboratory is described as a social environment in which participating actors carry out interventions in the form of ‘real-world experiments’ (RwEs) (Gross and Krohn 2005; Schneidewind 2014). Schneidewind et al. (2018) highlight the structural components of real-world laboratories, encompassing interpretative schemes (e.g., norms and identities), legitimization rules, allocative (financial, personal), as well as authoritative resources. As such, they constitute a structural context that enables agency and, vice versa, they are also shaped by the agentic activities of scientific and societal actors (Giddens 1984). Real-world laboratories involve both structural and procedural dimensions including joint definition, experimentation, learning, and implementation which increases the relevance and value of research (Hirsch Hadorn et al. 2008; ; Schneidewind et al. 2011; Jahn et al. 2012; Bergmann et al. 2021). Experiments support transdisciplinary integration (cognitive, social, communicative) and enable the direct, flexible, and small-scale implementation and evaluation of proposed solutions (Bergmann et al. 2021; Kampfmann et al. 2023).

Considering the challenges of involving young people in regional sustainability transformation (RST) processes on the one hand and the potential of real-world laboratories as structural context on the other, this paper aims to address the following research questions: (a) Which methods and tools support transdisciplinary knowledge co-creation with young people? (b) How can the structural conditions of the real-world laboratory enable young people’s participation in RST processes in the context of a RwE? (c) What are the effects of integrating young people in RST through a RwE?

This paper focuses on the results and limitations of the eight-month RwE ‘Youth Participation' conducted within the transdisciplinary research project 'Empowerment, Self-Organisation, and Regional Transformation—the Model of the Club of Rome Region Carnuntum' (RLC 2040) in the federal province of Lower Austria (Austria). Based on the five criteria defined by Bergmann et al. (2021), our 42-month RLC 2040 project qualifies as a real-world laboratory. This transdisciplinary project offered a comprehensive research infrastructure (Schneidewind et al. 2018), enabling experiments as a research methodology. Data are drawn from accompanying research and various methods employed during the RwE (see section "Materials and methods"). We critically assess our experiment experience and draw generalizable lessons for engaging young people in RST processes, encompassing the appropriateness of diverse methods and tools, structural conditions, and overall effects of this collaborative effort.

The paper follows this structure: Section "State of the art and conceptual framework" reviews the current state of youth participation and real-world laboratories. Section "Materials and methods" outlines the case study, the methodological approach, and experiment process. Section "Results" presents our findings, which are discussed in Section "Discussion", and leads to general conclusions.

## State of the art and conceptual framework

Transdisciplinary research involving youth highlights their active role as contributors to research quality (Camponovo et al. 2023; Moody et al. 2021). Youth participation models (e.g., Hart 1992; Shier 2001) focus on enhancing young people’s influence in decision-making processes and promoting their rights, fostering social engagement, and active citizenship, including educational political and social empowerment (Checkoway 2011; Botchwey et al. 2019). Lundy’s (2007) model outlines four chronological stages through space, voice, audience, and influence, each of which is crucial to the realization of young people’s participation rights. The stages of youth participation begin with space, which creates a safe environment to express opinions without fear. Voice values young people’s ability to articulate their views, supported by relevant information. Audience requires decision makers to actively listen to young voices. Finally, influence emphasizes the need for adults to consider these views in decision-making processes. This goes hand in hand with research emphasizing trust, respect, adult encouragement of ideas, and stable relationships as crucial for youth participation (Checkoway 2011; Gal 2017; Kennan et al. 2019). Successful engagement furthermore involves recruitment, voluntary participation, benefits, and privacy (Lund 2007; Lundy 2007; McCafferty 2017; Sarkadi et al. 2023) as well as age-appropriate communication and creative methods that resonate with youth interests and empower them to freely express their opinions (Gal 2017; Herzig Gainsford et al. 2017).

To raise awareness of ethical aspects of research with children, specific guidelines have been developed, such as the International Charter for Ethical Research with Children. The Charter commits the research community to uphold the highest ethical standards, to respect the dignity and rights of children, to ensure justice and fairness, to design research for the benefit of children, to prevent harm, to obtain informed consent from children, and to reflect continuously on ethical practices (Graham et al. 2013).

Engaging young people in meaningful problem solving fosters their civic engagement, relationships, and personal development (Checkoway 2011; Duncan et al. 2020; Siener 2021; Toros 2021; Bauml et al. 2022), while community identity has a lasting impact on their civic participation and identity formation (Bühlmann 2010; Siener 2021). Youth participation overcomes outdated views, fosters innovative projects, enhances community well-being and motivation, generates local youth knowledge, and legitimizes decision-making processes among them (Marini and Volk 2017; Botchwey et al. 2019; Siener 2021; Toros 2021; Bauml et al. 2022). The paper contributes to the discourse on appropriate methods and tools, structures, and the perceived effects of youth participation in RST.

### Methods and tools

Research indicates the need for diverse techniques to effectively engage young people from various backgrounds (Francis and Lorenzo 2002; Hill et al. 2004). In real-world laboratories, methods of collaboration between scientific and societal actors vary from providing information to intensive collaboration (Bergmann et al. 2021). Effective youth involvement in planning processes requires appropriate methods to ensure authentic participation (Francis and Lorenzo 2002), such as environmental autobiography (Francis and Lorenzo 2002), photo voice or gamification (Burke et al. 2017), diary methods, participatory drawing or photo diaries (Literat 2013), and participatory mapping (Dennis et al. 2009; Literat 2013; Cochrane and Corbett 2018).

Participatory mapping (PM) is a cartographic approach that supports communication and collaboration by visualizing the relationships between places and local communities (Cochrane and Corbett 2018). It enables young people to identify and discuss relevant issues using their local knowledge, promotes personal and social skills, and increases a sense of recognition and purpose (Tunstall et al. 2004; Dennis et al. 2009; Literat 2013). Key aspects of PM include using participants’ language to promote inclusivity and creativity (Franklin 2022), sharing information and dialogue (Gallagher et al. 2012; Gal 2017), and fostering relationships between young people and adults (Checkoway 2011). In addition, interactive environments that bring children of different ages and abilities together support greater learning opportunities than traditional school systems with strict age and ability segregation (Hart 2008).

In real-world laboratories, effective communication is essential due to the heterogeneity of participants and requires tailored communication formats to promote interaction, knowledge integration, feedback, and shared decision making (Bergmann et al. 2021; Fischer et al. 2024). The combination of information methods (articles, public information, mailings) and consultation methods (surveys, interviews, personal communication, lectures, and workshops) is particularly effective (Bergmann et al. 2021).

### Structural conditions

From a structuralist perspective, a real-world laboratory serves as an essential research infrastructure that mobilizes patterns of interpretation, norms, and resources for RwEs that contribute to reflection and reinterpretation and influence structural dimensions (see Table 1) (Schneidewind et al. 2018). Youth participation is highly dependent on sociopolitical structures and requires adapted governance structures for effective engagement (Gal 2017). The Lundy model (2007) emphasizes that it is not enough to have a voice; young people need to be able to communicate their views directly to decision makers who will not only listen to them but also take them into account in their decisions. Young people’s motivation increases in contexts where they can have a tangible impact (Haury 2014; Gal 2017; Herzig Gainsford et al. 2017; Bauml et al. 2022). Trust and stable relationships are crucial for engagement, alongside empowerment, ownership, motivation, organizational capacity, and leadership as key factors for success (Cochrane and Corbett 2018; Kennan et al. 2019; Bammer et al. 2020; Literat 2013).

**Table 1:** Structural dimensions of a real-world laboratory (Schneidewind et al. 2018)

Structural dimension	Purpose and definition
Interpretative schemes	For a common understanding of key concepts and terms For mobilization of actors based on local identity For a shared understanding of the problem
Legitimization rules	To share norms To justify the interference of actors
Allocative resources	For available human resources For available financial resources For the scope and depth of the transformation process
Authoritative resources	To use power to control political or organizational processes To ensure that all groups involved have a say To realize ideas developed

### Effects

Capturing the effects of transdisciplinary research is challenging due to the long-term and context-dependent nature of its scientific and societal impacts, making it difficult to attribute specific outcomes to particular projects (Schäfer et al. 2021). Schäfer et al. (2021) propose an assessment model that captures the temporal and spatial dimensions of effects (first, second, and third order) and characterizes different forms (see Table 2).

**Table 2:** Categories for the analysis of societal effects of transdisciplinary research (Schäfer et al. 2021)

Category	Effects
First order: occurs during the transdisciplinary project and within its geographical scope; directly observable and directly related to project activities and outcomes	Learning and capacity building Network formation Improving the situation Increase in reputation
Second order: are in the narrow temporal and spatial context of the transdisciplinary project; initiated during the project period, but their realization depends on external actors and factors	Continuation of activities in the project content Transfer to other spatial contexts
Third order: go beyond the temporal and spatial context of the transdisciplinary project; cannot be directly attributed to specific project activities, as the project is only one of many influencing factors	Influence on public discourse New concepts Influence on legislation and regulation Further structural effects

## Materials and methods

### Case study—RwE ‘youth participation’ in the municipality of Fischamend

**Fig. 1:**
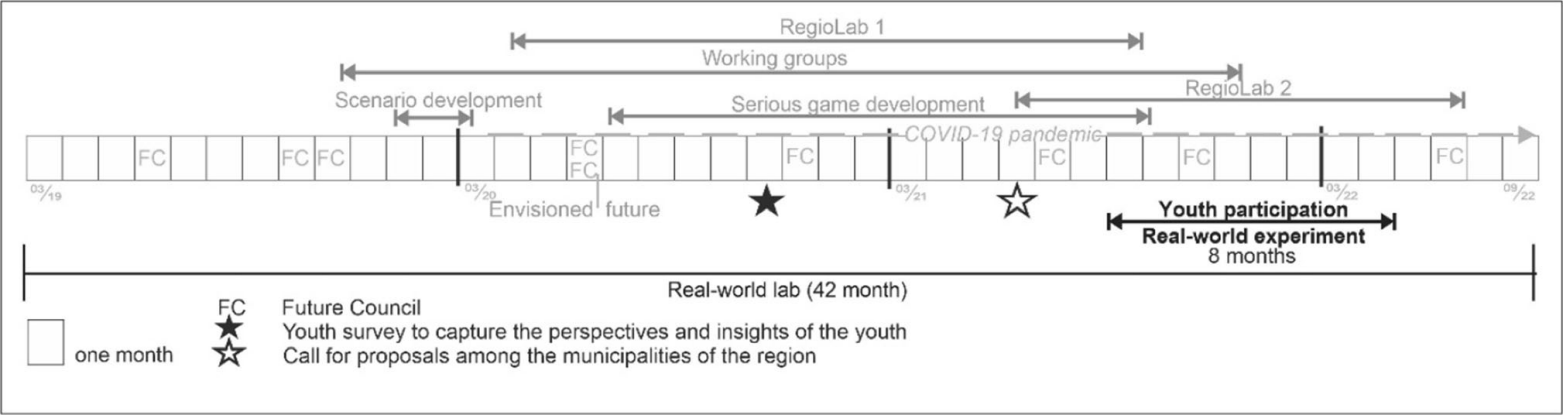
Embedding the RwE in the real-world laboratory RLC 2040 (own illustration)

The 42-month real-world laboratory *‘*Empowerment, Self-Organisation, and Regional Transformation—the Model of the Club of Rome Region Carnuntum' (RLC 2040) was implemented in the Römerland Carnuntum region (in the federal province of Lower Austria, Austria). This region encompasses a total of 30 municipalities and is located between the major urban centers of Vienna (Austria) and Bratislava (Slovakia). Collaborating with the Regional Development Association Römerland Carnuntum and two Austrian universities (University of Natural Resources and Life Sciences, Vienna, and TU Wien), the project aimed to (i) foster dialogue and establish a shared vision for the region’s future, (ii) facilitate deliberation and knowledge integration within the regional Future Council, serving as an advisory board and source of recommendations, (iii) experiment with innovative learning and participation methods, including scenario development, a serious game, RwE, and (iv) harnessing communication tools to enhance integration processes among actors (see Fig. 1). For further information see Radinger-Peer et al. (2022).

Ninety members were nominated for the Future Council, including 30 randomly selected citizens and 60 specially nominated representatives from municipalities and the Regional Development Association. For further information, see Gugerell et al. (2023). In addition, the transdisciplinary project team, consisting of scientists, practitioners, and integration experts (see Fischer et al. 2024), was an integral part of the Future Council. The selection process aimed for regional distribution, considering factors such as gender and age. In the end, despite many efforts, only 3% of the members were under the age of 19. The low participation of young people in the real-world laboratory led to a RwE to better involve them in regional future issues in a co-creation process.

As an initial step, a survey of young people in the region on the vision developed as part of the real-world laboratory (November 2020) (see Figs. 1 and 3) revealed a need for *‘*public places and open spaces where young people can meet.’ This rather specific aspect of the quality of life of young people in the region is seen in the broader context of their regional identity and belonging, embeddedness in regional networks, and participation in decision making, all of which influence the potential and processes of RST.

The Future Council appealed to the municipalities in the region to find a place for a youth participation experiment. Fischamend, a municipality with approximately 5700 inhabitants (as of 2023), on the southern outskirts of the city of Vienna, responded to this call.

**Fig. 2:**
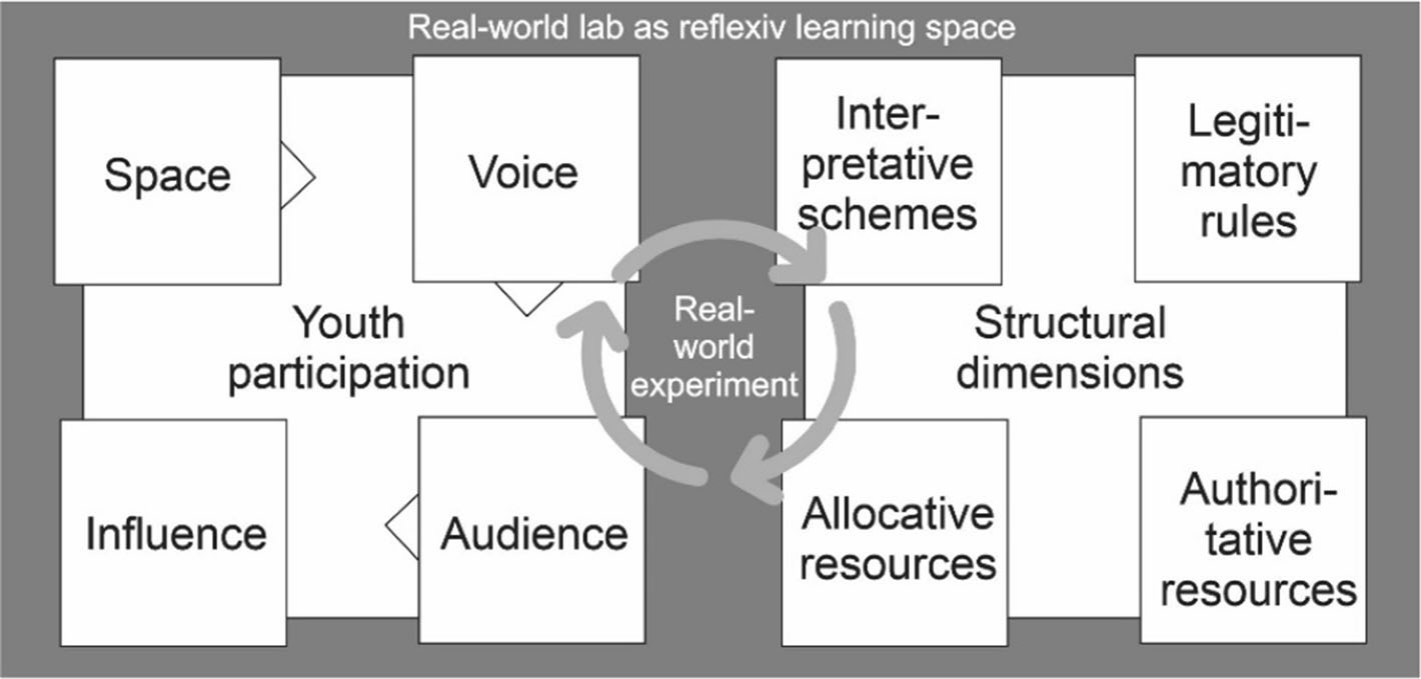
Dimensions of youth participation and structures in the RwE ‘Youth participation’ Fischamend (federal province of Lower Austria, Austria) (own illustration according to Lundy 2007 and Schneidewind et al. 2018)

The RwE ‘Youth Participation’ was devised by a transdisciplinary project team consisting of scientists, a community representative, and a youth worker of the municipality. The collaborative process involving young people and community representatives emphasized the input of young people. Structural components of real-world laboratories according to Schneidewind et al. (2018) were applied (see Fig. 2):

- *Interpretative schemes* the joint definition of the main concerns and problems for young people in the Römerland Carnuntum region, identified as a lack of quality of life due to missing public places and open meeting areas, formed the starting point for the experiment.
- *Legitimization rules* the regional development agency and, as part of it, the youth workers, but also the political representatives of the municipality lent legitimacy to the RwE.
- *Allocative resources* the transdisciplinary research project provided personal support for the implementation of the RwE, and the community representatives invested their own personal and time resources; financial support was provided by the LEADER funding scheme of the region and the municipality’s resources. In addition, the premises of the youth center in Fischamend were made available for the organization of workshops, presentations, and meetings.
- *Authoritative resources* the mayor of the municipality of Fischamend as well as the youth councillor relinquished some of their authority and supported a shared decision-making process between youth and the local government. Furthermore, the interested young people were invited to participate in the detailed planning of their idea and its subsequent implementation.

**Fig. 3:**
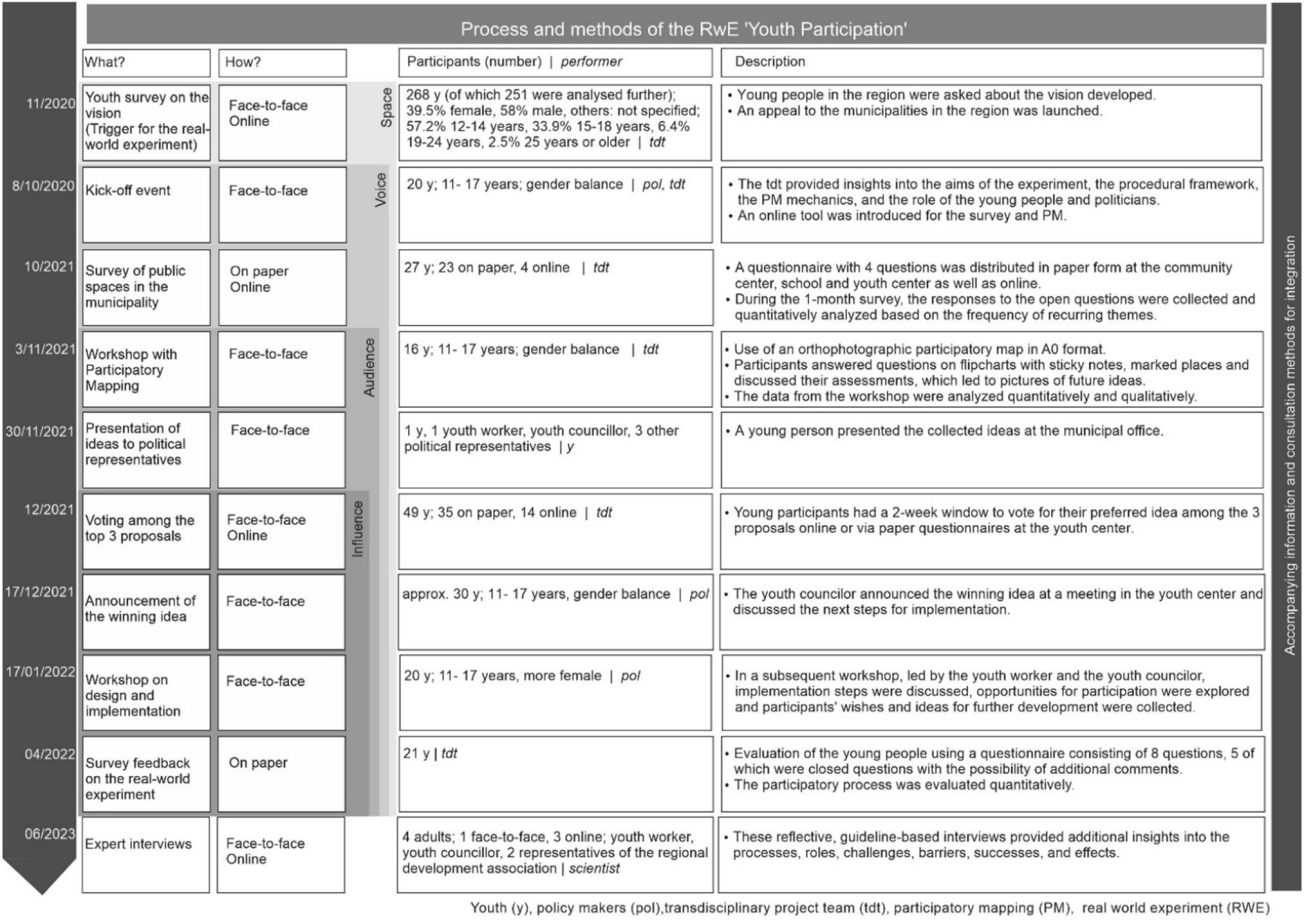
RwE ‘Youth Participation’ in the municipality of Fischamend (federal province of Lower Austria, Austria) (own illustration)

Overall, the structure of the RwE described below was set up experimentally and promised to be innovative and insightful (see Fig. 3).

### Methodological approach and RwE

By using a variety of methods, activities, and communication tools that were helpful for integration, the RwE aimed to identify young people’s needs, cultivate a learning community involving youth, the youth worker, and local policymakers, and develop shared knowledge and joint solutions (see Fig. 3).

In consultation with the Future Council, PM was chosen as the method for the RwE because it was suitable for this age group (at least 11 years) visualized spatial issues on maps, and could be carried out flexibly online and in person. Information methods (letters, newsletters, information events, posters, homepages, kick-off event, announcement of the winning idea) and consultation methods (surveys—partly with PM, interviews, workshops—one with PM, voting cards, personal communication) were also used.

At the beginning of the RwE, the youth councillor sent personalized letters to 350 young people aged 12–18 in the municipality, inviting them to participate in the experiment and explaining the methodological approach. Posters with QR codes linking to special online information pages announced the kick-off workshop and the RwE.

As can be seen in Fig. 3, PM was presented and explained at the kick-off event. In the subsequent questionnaire, participants were able to mark specific suggestions for improvement on an integrated map of the municipality, indicating places to avoid, meeting points, attractive areas, and other suggestions.

The implementation of PM required methodological decisions on map formats (paper or digital), working methods (individual or group work), and the choice between printed maps and freehand drawings. The online tool used, 'Adhocracy,' is an open-source software specifically designed for online participation. The associated web pages featured a chronological timeline and a community map for zooming and marking locations. The pages provided comprehensive information about the overall process, the individual steps, contact details for surveys, and the presentation of results. Participation necessitated registration under a chosen username and a non-publicly visible email address. Information about the website was disseminated via posters, the youth councillor communications, and the municipality’s website.

By using a low-threshold approach in familiar surroundings and with the youth worker as a trusted person, the PM workshop promoted active participation, well-being, and the reduction of inhibitions. Participants identified their daily routes and key points, traced these on maps, and answered the same questions as in the first questionnaire. This created images of prospective future scenarios linked to their experiences.

A young person presented the collected ideas to the political representatives at the municipal office, who then selected three financially feasible proposals. The young people voted for their favorite, which the youth councillor announced at the youth center. In a subsequent workshop, concrete implementation steps, opportunities for participation, and participants’ wishes and ideas for further development were worked out. At the end of the RwE, this was assessed by the youths and evaluated by the scientists.

Fourteen months after the experiment, four expert interviews were conducted to provide additional insights into the processes, roles, challenges, barriers, successes, and effects.

## Results

### Methods and tools: how were the youth reached and motivated?

**Fig. 4:**
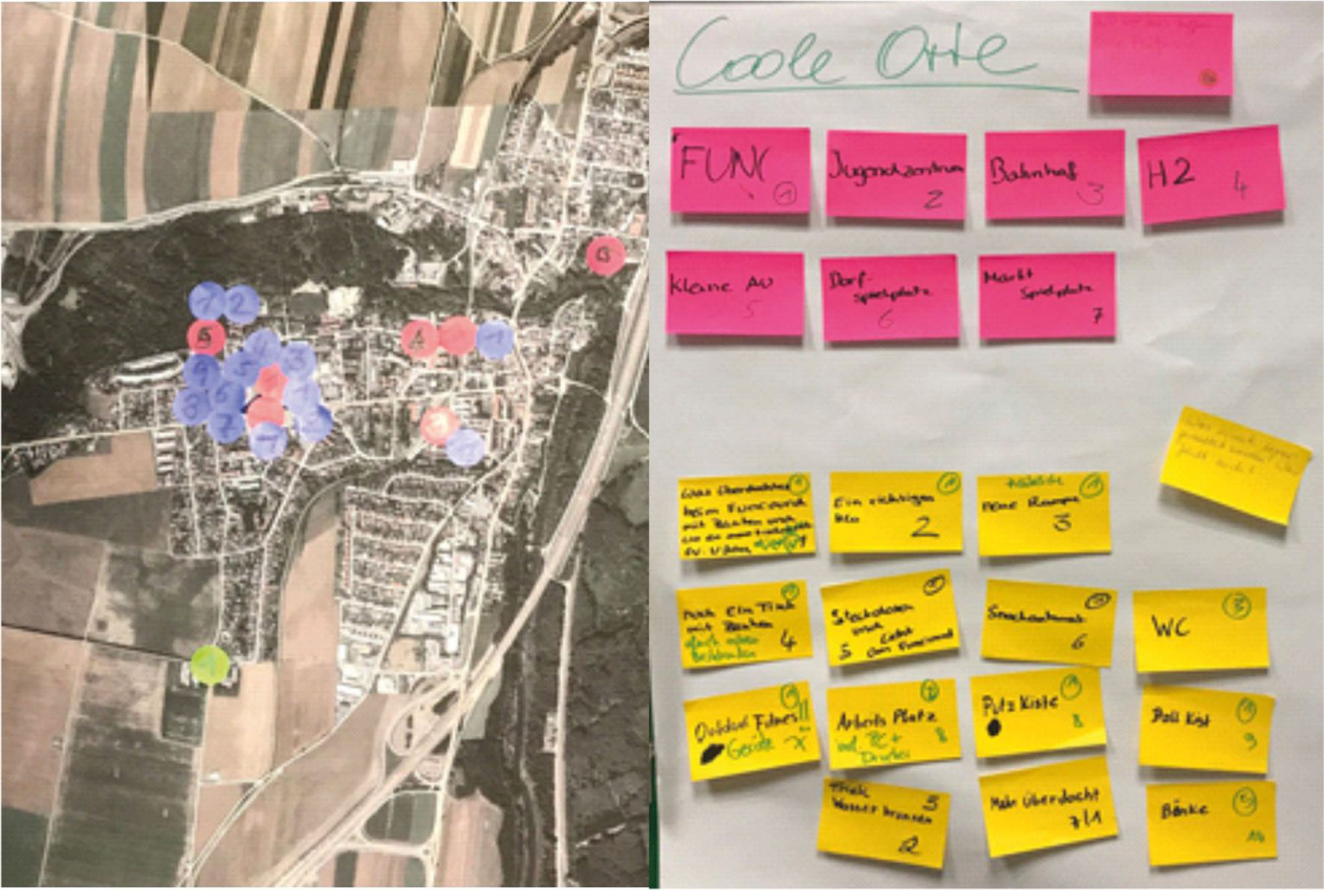
Results at marked locations on the community map and suggestions on sticky notes for a queried area in the workshop with PM

In the RwE, a mix of information and consultation methods was used, with PM as the primary technique to help children develop and express their opinions. Maps in the questionnaires should have enabled the young participants to record their opinions during the survey on public spaces both on paper and online and in a PM workshop. These maps were hardly used in the questionnaires. A total of 27 youths participated in the survey, with four engaging online (see Fig. 3). The survey results included four mentions of places to avoid, 26 of meeting spots, 26 of popular areas, and 33 of potential enhancements. The PM workshop at the youth center, attended by 16 youths aged 11–17 years and an equal split of genders, was conducted in a familiar environment and led by a well-known youth worker; a safe space was created. The lively and informal discussion dynamically evaluated the practicality of the proposals, which ranged from the purchase of a printer for the youth center to the improvement of the self-management of the funcourt with a lockable cleaning box. The youths’ proposals focused on tangible improvements and feasible ideas. The process (see Fig. 4) identified one place that young people avoid, seven cool places, and 19 suggestions for improvement, 15 of which related to the funcourt. In their feedback on the workshop, youth participants said that ‘it's great that the mayor wants to know what I think,’ but that they hope ‘our ideas will be implemented.’

In the voting involving 49 youths (14 online), a pavilion construction at the funcourt was selected as the top idea, aimed at enhancing community life for young people.

In the final workshop on design and implementation organized by the youth councillor and the youth worker, 20 young people participated to express their preferences for implementing the winning idea. The youth councillor communicated that implementation was assured, albeit with delays due to grant-dependent funding, which was met with both understanding and disappointment.

In the survey feedback on the RwE conducted on paper at the youth center, 21 young people participated, all of whom supported the experiment. Nineteen participants valued being consulted and contributing ideas; ten felt taken seriously as young people; eight appreciated that concrete actions were implemented and that leisure activities were enhanced. Seventeen found the youth participation successful, and all endorsed further youth involvement in municipal planning. Suggestions for improving engagement included more surveys at the youth center, using platforms like TikTok and Instagram, and above all, direct conversations with young people.

The personal letter informing young people about the RwE was highly effective, as evidenced by significant feedback to the youth councillor: ‘I was asked about it by young people on the street.’

The presentation of the collected ideas to the political representatives by a young person impressed them.

In the expert interviews and in informal discussions, the adults emphasized the importance of the many personal conversations with young people that developed in and outside the experiment. The youth worker stressed that ‘the personal approach is the most important tool. You have to talk to the young people, tell them that they can take part and that they can benefit from it.’ The online tool used was viewed critically,’although in principle I am enthusiastic about it’ (youth councillor); the youth worker and the youth councillor criticized the need to provide an email address, the double-opt-in procedure, and the lack of mobile phone friendliness of the tool. They pointed out that the majority of young people tend to carry out activities on their mobile phones rather than on computers and recommended the use of more familiar tools.

### Structural conditions: who and what is needed to involve the youth?

The RwE was launched by a youth survey, which highlighted young people’s concerns and problems, and provided a common basis for further activities in the experiment. *Interpretation schemes* were initially developed within the transdisciplinary project team and then extended to include policymakers, and young people, facilitated by regional youth workers who played a key role in building mutual understanding. Their contribution to the development of the questionnaires, the coordination of the surveys, and the organization of the workshops contributed to a common understanding of local identities. Scientists and societal actors worked together on an equal footing and the scientists used comprehensible methods and everyday language. The process allowed political representatives to receive direct feedback from young people, thus promoting learning opportunities from RwE.

Transparency in communication about clearly defined objectives, processes, decision making, expectations, and funding of the RwE contributed to the development of *legitimacy structures*. Respect for ethical and social rules was crucial. Young people always had the opportunity to choose their participation and could leave the workshops at any time. Cooperation with the municipality was agreed on the condition that the results would be implemented and communicated to the young people as a benefit from the outset. Anonymous data collection allowed young people to express their opinions openly but limited the precision of the sample survey. The youth center served as a safe space and the youth worker as a trusted reference person. The use of scientific methods, the transparent handling of results, and a mutual decision-making process between young people and political representatives, which was part of the wider RLC 2040 real-world laboratory, and the involvement of the established Regional Development Association strengthened the legitimacy of the experiment.

As agreed at the beginning of the RwE, a*llocative resources* were secured through human resources from the Regional Development Association and the municipality, complemented by scientific resources from the real-world laboratory. The municipality’s comprehensive support, endorsed by political backing, facilitated the prompt and efficient fulfillment of commitments to the young people.

In terms of *authoritative resources,* the active participation of the youth councillor, including support from the local political leadership, was of great importance*.* In the expert interview, the youth councillor explained his role as a driving force, supported by the mayor and the city council, who gave him the necessary room to carry out the experiment according to his ideas and to guarantee the implementation of the proposals. Channels such as the municipal website, notices, and the municipal newspaper were used for public communication, supplemented by the mayor’s social media posts.

### Effects: what was the added value of youth involvement?

*First-order effects.* According to the interviews, a learning process took place as part of the RwE, including awareness raising, skill building, and empowerment of all participants. Young people experienced that their opinions could bring about important changes, which increased their interest in continuing to participate in community planning processes. In the final survey, all participants were in favor of continued involvement in planning processes and valued specific community improvements. A trusting relationship between the youth councillor and the young people led to an increased assumption of responsibility by the young people for municipal infrastructure (according to the expert interview with the youth councillor). Expert interviews highlighted organizational learning and the positive impact of youth participation, with decision makers praising the feasibility and realism of youth proposals. Some suggestions, such as new basketball hoops and a cleaning box for the fun court, were implemented immediately, increasing young people’s confidence in the political process. The early involvement of young people was seen as formative for their future contributions and highlighted the role of youth work in regional development (expert interviews): *‘*Other youth workers asked how to do such surveys`. The implementation of youth ideas improved community relations and the reputation of the youth councillor, while the municipality attracted attention and positioned itself as a role model by sharing the experiment on social media and at regional events. All those involved could imagine organizing a participation process with young people again.

*Second-order effects.* The implementation of the winning idea within the framework showed the limited time and space context of the RwE. Even after the project, the youth councillor and the Regional Development Association acted as knowledge brokers for other municipalities in the region, which in turn showed interest in implementing similar participation projects, whether with young people or adults. The strengthening of the youth councillor’s role was reflected in increased requests for advice on successful youth participation and his presentation of the process at various events: *‘*Many municipalities contacted me afterward, and we got the ball rolling.’ In addition, the importance of participation was embedded in the newly elaborated development strategy of the Regional Development Association. The successful implementation of youth participation in the municipality of Fischamend has established the municipality as a pioneer and model for other municipalities.

*Third-order effects.* The project contributed to the transformation of the regional public discourse on youth participation and it influenced the adaptation of framework conditions in the region. A representative of the Regional Development Association said: *‘*I will take that with me: I am sure, youth work is important for regional development. If you feel recognized as a young person, you will get involved as an adult.’

## Discussion

The implementation of the RwE 'Youth Participation' in Fischamend municipality (in the federal province of Lower Austria), aimed to explore youth involvement in RST processes, triggered by their low participation in the real-world laboratory RLC 2040.

Our findings reveal and confirm five crucial points for integrating young people in RST via a RwE approach: (1) a concrete/specific topic, in a context that is relevant to young people and where they can make a difference (Gal 2017); (2) a trusted intermediary, in our case the youth worker, who translates between the young people and the scientists as well as local policymakers (Fischer et al. 2024); (3) legitimacy of the youth participation process, via support from political decision makers; (4) favorable structural conditions of the RwE including human and financial resources from the beginning to the implementation (Schneidewind et al. 2018); (5) shared decision making between young people and local policymakers, where young people’s opinions are heard and given due weight (Lundy 2007).

In addition, our results emphasize the importance of political representatives dedicating time and attention to youth, that young people prefer face-to-face communication, and that PM is effective in familiar contexts with well-known people, also referred to as ‘safe space.’

Sarkadi et al. (2023) emphasize the importance of the methods used to recruit and involve young people in research processes. Participation in surveys and workshops was both anonymous and voluntary, and participants were informed in advance about these conditions and how their data would be handled. All adults involved were aware of their responsibility toward the young people and followed the ethical and social rules. In addition, the youth worker continuously emphasized that the focus must be on the perceived benefits and influence of youth. Thereby it was important for the regions political representatives to recognize the value of youth participation without viewing it as a complex technical process which requires special skills (Lundy 2018); out of five potential municipalities, however, only one has dared to implement a RwE. Expert interviews revealed that politicians were impressed by the youth’s realistic and unexpected proposals as well as their capability of rational decision making (Botchwey et al. 2019), challenging and diminishing outdated perceptions of the youth (Siener 2021). In addition, the youth center offered a safe, extracurricular environment that ensured that the young participants did not have to fear reprisals (Lundy 2007).

The findings affirm that effective participation requires specific issues relevant to young people’s lives and adult support to encourage their engagement (Checkoway 2011; Gal 2017). In the real-world laboratory envisioning the region’s future to 2040, efforts to engage young participants were in vain. The focus on a concrete and feasible topic stimulated active youth involvement in the project (Siener 2021; Bauml et al. 2022). The RwE in the municipality was conditional on the young people’s preferred idea being implemented. From the outset, young people were informed that their contributions would influence decisions and the importance of their active participation and power sharing in decision-making processes was emphasized (Shier 2001; Lundy 2007).

In the context of RwE, the youth worker from the municipality of Fischamend played a central role as a contact person, bridging person, and intermediary between the young people and scientists (Bammer et al. 2020; Radinger-Peer et al. 2022; Fischer et al. 2024). This role involved translating interests and co-creating 'interpretative schemes' as common norms and narratives in the process. This role, coupled with building personal relationships and providing support outside institutional frameworks, enhanced youth participation. The established trust encouraged youth to engage with the youth worker beyond organized meetings to discuss their ideas and the community participation process (Checkoway 2011; Literat 2013; Gal 2017; Bauml et al. 2022). However, he also encouraged the young people to get involved, pointing out that they could have an influence (Lundy 2007) and act as a point of contact for the youth councillor.

As pointed out by Schneidewind et al. (2018), a real-world laboratory can be understood as an experimental space that interacts with structural conditions at the regime level to change them, although little is yet known about the influence of the structural components of these laboratories on the overall process. We aim to contribute to this literature by reflecting on the findings from the RwE ‘Youth Participation’ from this perspective. As already mentioned, the joint definition of the problem (Hirsch Hadorn et al. 2008) based on the region-wide survey of young people was essential. A further success factor, belonging to Schneidewind et al.’s (2018) legitimization rules, was the embedding of the RwE in the real-world laboratory RLC 2040. The establishment of trust among scientific actors, municipal officials, and young people (Literat 2013), combined with the active participation of the youth councillor and support from Fischamend’s local government, enhanced the legitimacy of the RwE (Bauml et al. 2022), were pivotal for the success of the process, legitimizing it for young people (Marini and Volk 2017). In addition, the real-world laboratory provided personnel and financial resources, including funding from the LEADER scheme in the Römerland Carnuntum region and municipal funds. These context-dependent favorable allocative resources (Gal 2017; Schneidewind et al. 2018) enabled the implementation of the RwE and the realization of the winning idea. Regarding the latter, the shared decision-making approach as part of the overall governance of the RwE enabled young people to have space, voice, audience, and influence and to make a difference (Lundy 2007; Haury 2014; Herzig Gainsford et al. 2017; Gal 2017). The RwE participants, aged 11–17 years, not only would like to see the winning pavilion idea realized but also wanted to be sure that it would meet their needs. The younger participants expected to benefit from the pavilion for a long time, while the older ones took on responsibilities such as maintaining the cleaning box.

Various creative methods such as the scenario process and serious gaming were used as part of the real-world laboratory, but these did not reach the youth. Given this, the transdisciplinary project team decided to try out an alternative method in connection with a specific topic. The RwE used diverse tools and methods, including PM in both paper and online questionnaires and workshops. Maps were underutilized in the questionnaires, but the PM workshop was particularly effective in generating ideas, demonstrating not only the influence of method choice on outcomes (Lansdown 2014; Camponovo et al. 2023) but also the need for co-creation. Initially, the young participants had difficulties finding their way around the community map during the workshop. Their engagement and orientation improved considerably when they were asked to identify places in their daily environment on the map and to trace their daily routes. PM facilitated discussions about public space, with the PM workshop generating extensive suggestions and enjoyable discussions, indicating the efficacy of maps for collaboration and communication (Dennis et al. 2009; Literat 2013). Instruments that facilitated communication between young people, the project team, and local politicians (e.g., presentations including discussion, and votings) and at the same time promoted transdisciplinary knowledge building supported the RwE (Fischer et al. 2024). As the feedback from the RwE survey showed, what young people wanted most was a personal conversation and being asked personally. The online tool was used less than the paper surveys. We attributed this to the personal approach of the youth worker, who drew the young people’s attention to the paper questionnaires available at the youth center. In addition, technical barriers to the online tool, such as a double-opt-in process, the need for a separate email address, and limited mobile usability, contributed to lower usage. Young people said they would like to use online tools in the future, but only those they are familiar with (TikTok, Instagram). Elements such as the joint decision-making process, the rapid implementation of the winning idea, and the PM workshop (Tunstall et al. 2004; Dennis et al. 2009) were perceived by the young people as promoting integration.

The effects of the RwE included universally positive assessments from participants, highlighting strengthened relationships between young people and political representatives (Bühlmann 2010; Checkoway 2011; Duncan et al. 2020; Siener 2021; Toros 2021; Bauml et al. 2022). These experiences enhanced young people’s self-efficacy and resulted in positive feedback from municipal representatives, creating a welcoming environment for their ideas within the municipality. The positive example of the municipality sensitized the entire region to the needs of young people and motivated several municipalities to think about (youth) participation processes. The youth councillor served as a regional multiplier, inspiring other municipalities to initiate participation processes with young people (Shamrova and Cummings 2017). Criticism was voiced at the regional level, pointing to difficulties in funding such processes outside of research projects. The young people extended their engagement beyond the RwE by taking their concerns directly to the councillor and actively shoulder responsibility for the implementation of their ideas (Frank 2006; Checkoway 2011; Shamrova and Cummings 2017; Botchwey et al. 2019; Siener 2021).

RwE helped to raise the awareness of young people throughout the region and to establish the issue of youth participation. Other municipalities in the region *‘*lost their fear’ (expert interview) of involving young people in regional transformation processes for sustainability, and political and regional decision makers learned which factors can support youth participation.

Limitations of the RwE stem from its project-based nature, where young people’s participation relies on adult and research project funding dynamics (Lansdown 2014). In addition, the RwE heavily leans on established youth work and collaboration between young people and youth workers (Bauml et al. 2022). The anonymous data collection limited a more precise description of the sample but offered greater protection for the young people.

## Conclusions

This paper aims to investigate the involvement of young people in RST processes using the example of the RwE 'Youth Participation' in the municipality of Fischamend (in the province of Lower Austria, Austria).

The quality of life of young people in a region is considered in the context of their regional identity and belonging, which is characterized by their involvement in regional networks and their participation in decision-making processes. These factors are crucial for the potentials and processes of RST. Despite our example focused on a short-term experiment aimed at pattern learning, the results indicate far-reaching effects that go beyond the RwE—in terms of time, space, and topic. The RwE has shown that young people are capable of making rational decisions and actively participating in RST processes.

The experience of RwE in the municipality of Fischamend showed that success depends on (a) the process of participation, including the methods to enforce collaborative learning and (b) the structural conditions, including trust persons (e.g., the youth councillor and youth worker in our example), a safe space, decision-making power, and a specific topic (interpretative scheme) which awakens interest. Besides revealed first-order effects such as social innovations regarding the role of youth in RST in the case study region, second- and even third-order effects can be detected. The positive experiences of the case study municipality inspired a certain degree of mimetic behavior in other municipalities in the region, which introduced youth participation processes. How these involvements as young inhabitants affect later participation in RST processes as well as regional identity and belonging as adult could be a desirable focus of long-term accompanying studies.

## Footnotes

1 The terms *‘*children’, *‘*child’, *‘*young people’ and *‘*youth’ are used interchangeably in this article and are meant to convey the definition found in UNCRC (1989) Article 1 which states ‘a child means every human being below the age of eighteen years’.
